# Identification of Yellow Advanced Glycation End Products in Human Skin

**DOI:** 10.3390/ijms25115596

**Published:** 2024-05-21

**Authors:** Bin Fang, Lijuan Li, Jason Winget, Timothy Laughlin, Tomohiro Hakozaki

**Affiliations:** The Procter & Gamble Company, Mason Business Center, Mason, OH 45040, USA; fang.b@pg.com (B.F.); li.l.28@pg.com (L.L.); winget.jm@pg.com (J.W.); laughlin.lt@pg.com (T.L.)

**Keywords:** skin yellowness, glycation, advanced glycation end products (AGEs), (1R, 8aR) and (1S, 8aR)-4-(2-furyl)-7-[(2-furyl)-methylidene]-2-hydroxy-2H,7H,8AH-pyrano-[2,3-B]-pyran-3-one

## Abstract

Skin yellowness is a hallmark of dull or unhealthy skin, particularly among Asians. Previous research has indicated a link between skin glycation and skin yellowness. However, the specific glycated chemicals contributing to yellowish skin appearance have not been identified yet. Using HPLC-PDA-HRMS coupled with native and artificially glycated human epidermal explant skin, we identified intensely yellow colored glycated chromophores “(1R, 8aR) and (1S, 8aR)-4-(2-furyl)-7-[(2-furyl)-methylidene]-2-hydroxy-2H,7H,8AH-pyrano-[2,3-B]-pyran-3-one” (abbreviated as AGEY) from human skin samples for the first time. The abundance of AGEY was strongly correlated with skin yellowness in the multiple skin explant tissues. We further confirmed the presence of AGEY in cultured human keratinocytes and 3D reconstructed human epidermal (RHE) models. Additionally, we demonstrated that a combination of four cosmetic compounds with anti-glycation properties can inhibit the formation of AGEY and reduce yellowness in the RHE models. In conclusion, we have identified specific advanced glycation end products with an intense yellow color, namely AGEY, in human skin tissues for the first time. The series of study results highlighted the significant contribution of AGEY to the yellow appearance of the skin. Furthermore, we have identified a potential cosmetic solution to mitigate AGEY formation, leading to a reduction in yellowness in the in vitro RHE models.

## 1. Introduction

The human skin, in addition to protecting our internal physiological environment, plays a crucial role in presenting ourselves to the world. The visual perception of one’s skin can be a vital indicator of health, vitality, beauty, and well-being. The condition and appearance of our skin are inherently linked to our overall well-being and self-esteem [1]. A clear, even-toned skin complexion often contributes to positive aesthetic perception [2]. However, the skin may exhibit various discolorations attributed to natural biological processes, aging, lifestyle, and environmental stress. These discolorations can manifest as patches of lighter or darker skin, or even changes in the overall skin tone [3,4].

Among concerns regarding skin discoloration, transient and chronic skin yellowness have emerged as unpleasant skin issues, especially among Asian women, necessitating further scientific understanding to identify suitable approaches to mitigation. We recently identified bilirubin as a contributor to transient skin yellowness, particularly in cases of acute oxidative stress or sleep deprivation [5]. However, for chronic skin yellowness, despite recent scientific investigations that have provided insights into a potential link between skin yellowness and glycation, no specific yellow chromophores have been identified yet. [6,7].

Glycation, also known as the Maillard reaction, occurs when reducing sugars react with proteins, resulting in the formation of a wide range of advanced glycation end products (AGEs). To date, at least three dozen AGEs have been characterized, and approximately 20 AGEs have been detected in human skin [8,9]. AGEs in the skin can impact skin health and affect the appearance of skin tone. The formation of AGEs is facilitated by oxidative stress, and the formed AGEs can further stimulate the production of reactive oxygen species (ROS), thereby intensifying the vicious cycle of oxidative stress [10]. Oxidative stress can also induce the formation of reactive carbonyl species (RCS), such as glyoxal (GO) and methylglyoxal (MGO) which can lead to the formation of carbonylated proteins. An example of a protein carbonylation product is N^ε^-(Carboxymethyl)lysine (CML), which is also categorized as an AGE. Diabetic patients typically exhibit elevated levels of CML due to prolonged exposure to high levels of glucose and the subsequent glycoxidative stress.

Protein damage resulting from AGE formation can manifest as cross-linking, altered surface charge, and direct damage, all of which can affect the function, recycling, and interaction of proteins. The adverse effects of AGEs on proteins have been well demonstrated in diabetic patients and are associated with the development of various complications, including diabetic nephropathy, retinopathy, neuropathy, and vascular injury [11,12,13,14]. The accumulation of AGEs can also contribute to protein damage, which contributes to the aging process [15]. In the context of skin, excessive glycation can be attributed to unhealthy lifestyles, such as high-sugar diets, smoking [16], sun exposure [17], and poor sleep [18]. When combined with psychological stress, glycation can exacerbate protein damage [19], including collagen damage, which can result in a loss of elasticity [20]. In addition to the intrinsic health issues, the formation of AGEs can alter the esthetic properties of the skin. The direct formation of chromophores, fluorescence properties, and uneven skin surfaces may all contribute to the visual appearance, particularly in the case of skin yellowness [7]. These processes can occur even in young people, since AGEs have been detected in the skin tissues of people as early as in their twenties [7,21].

While the protein damage caused by AGEs has been extensively studied, the issue of yellowish discoloration associated with glycation in the context of skin has also received attention [7,22]. However, currently, there is a lack of in-depth studies specifically focusing on identifying and characterizing the yellow colored chemical(s) associated with glycation in the skin. Interestingly, well-known AGEs and carbonylated proteins identified in the skin, such as CML, fructoselysine, and pentosidine, are reported to be colorless [23]. For instance, CML has been a well-known biomarker for both glycation and carbonylation, however, it does not have a yellow color itself. Rather, its presence and level serve as an indicator of overall oxidative stress and signal the presence of other AGEs which may be yellow. In order to investigate the AGEs that contribute to the appearance of skin yellowness, we conducted a series of studies using in vitro and ex vivo skin models, along with state-of-the-art analytical tools. Additionally, we examined the effect of a combination of cosmetic compounds, which possess anti-glycation effects, on the production of yellow AGEs in 3D reconstructed human epidermal (RHE) models.

## 2. Results

### 2.1. Identification of Novel Yellow Chromophores (AGEY) from Human Epidermal Explant Skin Tissue

**Figure 1:**
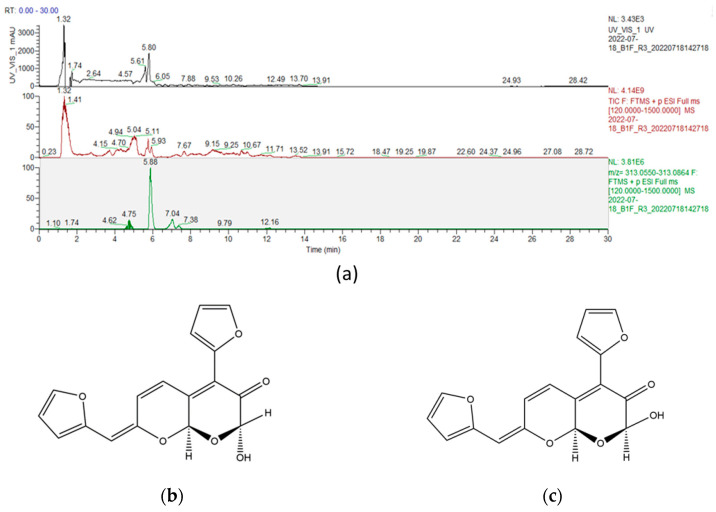
(**a**) Top panel: UV/Vis chromatogram of human epidermal explant hydrolysate at 440 nm; middle panel: total ion chromatogram (TIC) of human epidermal explant hydrolysate; Bottom panel: extract ion chromatogram (EIC) of human epidermal explant hydrolysate (*m*/*z* = 313.0707 at 5.88 min); (**b**) chemical structure of (1R, 8aR)-4-(2-furyl)-7-[(2-furyl)-methylidene]-2-hydroxy-2H,7H,8AH-pyrano-[2,3-B]-pyran-3-one; and (**c**) chemical structure of (1S, 8aR)-4-(2-furyl)-7-[(2-furyl)-methylidene]-2-hydroxy-2H,7H,8AH-pyrano-[2,3-B]-pyran-3-one.

From the hydrolysate of human epidermal explant punch biopsies obtained from three female individuals aged 50 (skin type IV), 56 (skin type III), and 61 (skin type III), we isolated compounds that exhibited an absorption peak at a wavelength of 440 nm, indicating a yellow color, using the UHPLC-PDA-HRMS technique (Figure 1a). The most prominent peak appeared at a retention time of 5.80 min, and the exact mass of this positively charged ion was 313.0707, which assigns a molecular formula of C_17_H_12_O_6_ to the compound. Through an extensive search of the literature, we were able to identify the lead molecules as “(1R, 8aR) and (1S, 8aR)-4-(2-furyl)-7-[(2-furyl)-methylidene]-2-hydroxy-2H,7H,8AH-pyrano-[2,3-B]-pyran-3-one” (Figure 1b,c) [24], abbreviated as “AGEY”.

**Figure 2:**
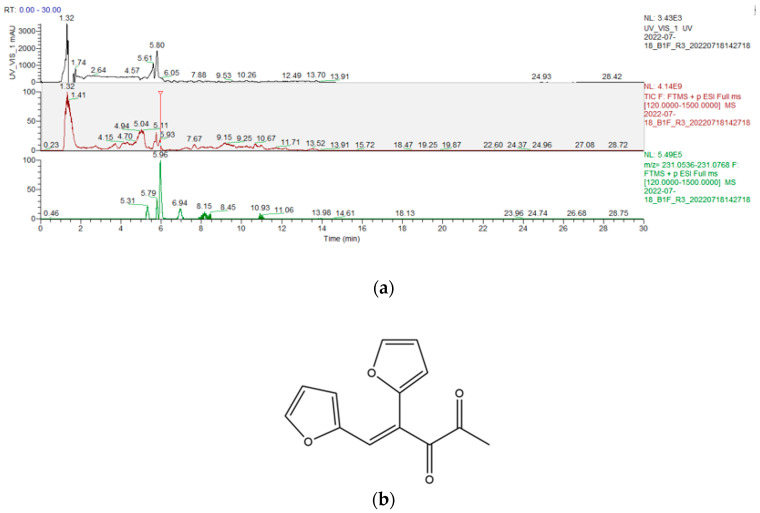
(**a**) Top panel: UV/Vis chromatogram of human epidermal explant hydrolysate at 440 nm; middle panel: total ion chromatogram (TIC) of human epidermal explant hydrolysate; bottom panel: extract ion chromatogram (EIC) of human epidermal explant hydrolysate (*m*/*z* = 213.0652) at 5.96 min); and (**b**) chemical structure of (4E)-4,5-di-2-furanyl-4-penetene-2,3-dione.

We also observed a minor peak at a retention time of 5.90 min. The exact mass of this positively charged ion was 231.0652, which assigned a molecular formula of C_13_H_10_O_4_ to the compound (Figure 2a). This matches the identification of the molecule, (4E)-4,5-di-2-furanyl-4-pentene-2,3-dione (Figure 2b). However, the signal intensity of this molecule is only a fraction of the AGEY signal and is considered negligible in level. We also examined other yellow colored AGEs reported in the literature [25] using the extract mass approach, but no significant signals were observed in the chromatogram.

### 2.2. Contribution of AGEY to Skin Yellowness in Human Epidermal Explant Skin Tissue

**Figure 3:**
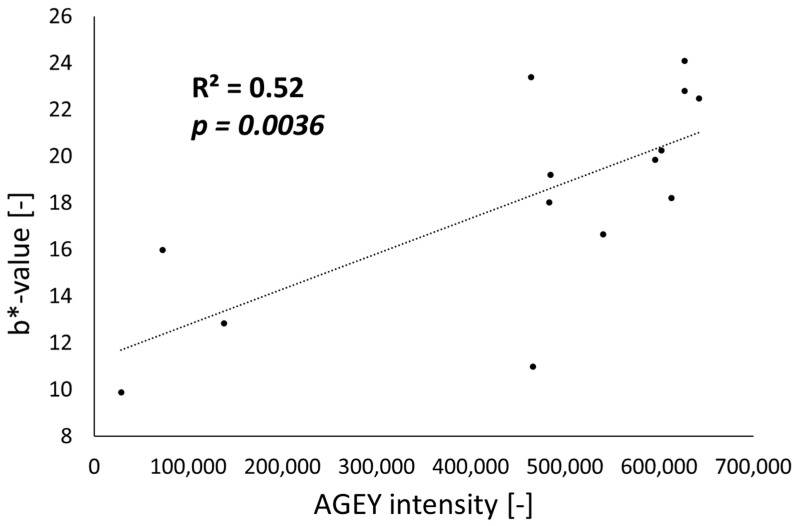
Correlation plot of relative AGEY intensity and skin tissue yellowness (b*-value) from 14 human epidermal explant tissues among the light skin group (Fitzpatrick type I-III). The *p*-value for the Pearson correlation coefficient was statistically significant (*p* < 0.05).

To verify the contribution of AGEY to the manifestation of skin yellowness, we quantified relative AGEY intensity, measured by the peak area, along with the measurement of the b*-value of skin explant tissues as a skin yellowness index. This analysis was performed on 14 human epidermal explant skin tissues (Fitzpatrick Skin Type I-III, age range—29–62 yrs, all females). We observed a strong positive correlation between AGEY intensity and b*-value (R^2^ = 0.52, *p* = 0.0036), providing confirmation that AGEY is a strong contributor of skin yellowness in human skin tissue (Figure 3).

### 2.3. Formation of AGEY with Glycation Reagent Treatment in Human Epidermal Explant Skin Tissue

**Figure 4:**
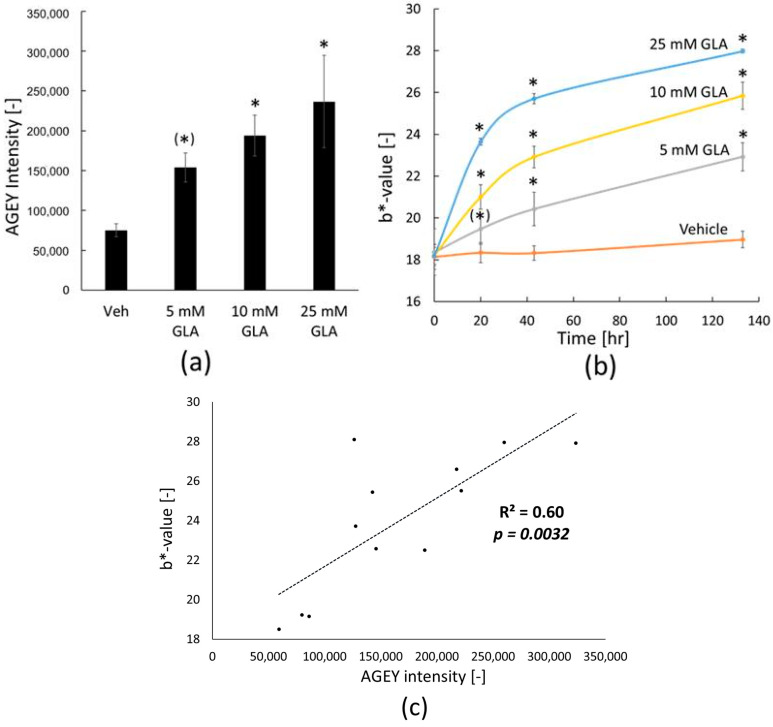
GLA treatment increases AGEY intensity as well as yellowness score (b*-value) in a dose dependent manner in human epidermal explant skin tissue. (**a**) Dose dependent increase in relative AGEY intensity quantified from GLA-treated human epidermal explant tissues. Treatment lasted for 133 h. Bar indicates mean ± SEM. n = 3 for each group. *; *p* < 0.05 vs. vehicle (*); *p* < 0.1 vs. vehicle (DPBS buffer) by ANOVA test of log10 transformed data followed by Dunnett’s multiple comparison test versus vehicle; (**b**) time course change in skin yellowness (b*-value) of human epidermal explant tissues treated with various doses of GLA. Plot data indicate mean ± SD. n = 3 for each group. * *p* < 0.05 vs. vehicle, and (*) *p* < 0.1 vs. vehicle for each time point using ANOVA test followed by Dunnett multiple comparison test versus vehicle; (**c**) positive correlation observed between skin tissue yellowness (b*-value) and AGEY intensity of GLA-treated human epidermal explant tissues. The *p*-value for the Pearson correlation coefficient was statistically significant (*p* < 0.05). Plotted data are from each individual tissue.

To determine whether AGEY formation is a result of glycation in skin tissue, we conducted an experiment in which human epidermal explant tissue was treated with DL-glyceraldehyde (GLA), a well-characterized glycation reagent. AGEY was detected in all skin samples treated with different doses of GLA in a dose-response manner (Figure 4a), indicating that AGEY formation can be induced by GLA treatment. Additionally, the GLA-treated explants exhibited a significant increase in skin yellowness (b*-value) at the end of the treatment (Figure 4b), and the increase occurred in a dose-dependent manner. The plot of individual sample data demonstrated a significant positive correlation (R^2^ = 0.60, *p* = 0.0032) between AGEY intensity and yellowness measurements (b*-value) among tested tissue samples (Figure 4c), also indicating the strong contribution of AGEY formed by glycation to the yellow appearance of skin tissues.

### 2.4. Detection of AGEY from Cultured Human Keratinocyte Cells

**Figure 5:**
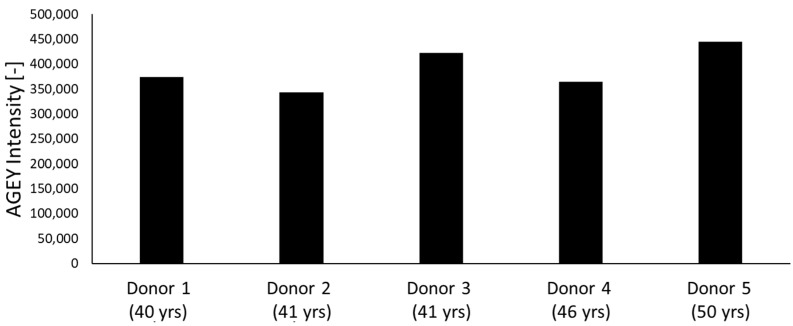
Relative intensity of AGEY in 5 cultured human primary keratinocytes from donors of various ages, measured by using HILIC-MS/MS technique.

To further understand the presence of AGEY at the cellular level, we investigated to determine if the AGEY identified in human explant skin tissues could also be detected in monolayer human keratinocyte cell cultures. As anticipated, we were able to detect AGEY in all five tested primary keratinocytes from different donors (donor age range: 40–50 yrs). This finding confirms that AGEY is not only present in skin tissues, but also present in cultured human keratinocytes (Figure 5).

### 2.5. Selection of Anti-Glycation Compounds Using Chemical Glycation Model

We also explored whether the formation of AGEY could be prevented by using cosmetic compounds with anti-glycation properties. To accomplish this, we developed a simple chemical glycation model using gelatin as a protein substrate and GLA as a glycating reagent. We employed a fast fluorescent intensity measurement as a marker of glycation degree, with aminoguanidine hydrochloride serving as a positive benchmark compound [26,27]. With this platform, we selected four distinct cosmetic ingredients, including chemical compounds and natural extracts (Table 1). Additionally, we assessed the combination of all four ingredients, which exhibited the strongest anti-glycation activity in this chemical glycation model.

### 2.6. Effect of Anti-Glycation Materials to Reduce AGEY and Skin Yellowness in 3D RHE Model

**Figure 6:**
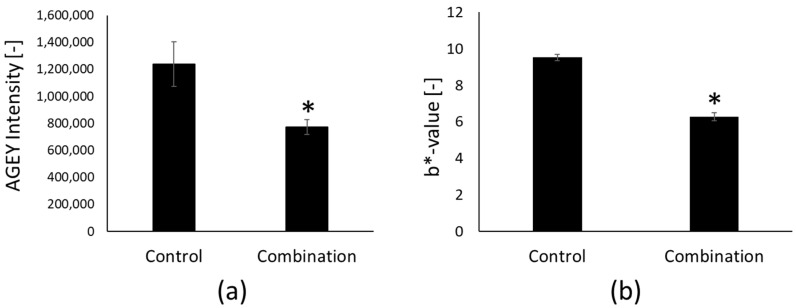
Effect of combined cosmetic ingredients on AGEY formation and skin yellowness in RHE model with 0.5 mM GLA treatment. A combination of 4 cosmetic ingredients (5% niacinamide, 0.1% lactobionic acid, 0.4% white water lily extract, and 0.1% artichoke leaf extract) were treated for 24 h in the medium and AGEY intensity and b*-value (yellowness index) of the RHE model were measured. (**a**) Relative AGEY intensity detected using HILIC-MS/MS technique; and (**b**) yellowness score (b*-value) measured by colorimeter. Bar indicates mean ± SEM. n = 5 for control; n = 4 for combination group; * *p* < 0.05 vs. control.

To examine the effect of the selected anti-glycation ingredients on AGEY formation and skin yellowness without interference from melanin chromophores (which can also affect skin yellowness), we utilized a commercially available RHE model without melanocyte (EpiDerm^TM^, MatTek). To observe the most pronounced effect, we tested the combination of the four selected anti-glycation materials with GLA treatment. We observed a statistically significant reduction in both relative AGEY intensity (Figure 6a) and skin yellowness (b*-value) (Figure 6b) compared to the control group.

## 3. Discussion

In the past few decades, there has been extensive research on the relationship between glycation and aging or skin health. In the context of skin, it has been hypothesized that accumulated AGEs contribute to visible signs of skin aging, including wrinkles and yellowish skin. Interestingly, recent research has revealed that skin glycation can occur in the epidermis even among young people and cause the appearance of yellowish skin [5]. This indicates that the early formation of yellow colored AGEs can occur in the epidermis even among young people. Interestingly, despite the extensive research on AGEs in recent decades, it has not been reported which specific structured glycation compounds are responsible for the appearance of yellow skin. Our recent consumer research in 2023 indicated that more than 40% of women globally reported skin dullness as an issue, with over 55% of women in China specifically describing it as yellow skin, highlighting the prevalence of the concern and the need to understand the specific glycation compounds that contribute to yellow skin appearance and develop effective intervention approaches.

In our investigation to determine the source of chronic yellowish skin appearance, we adopted native human skin explants as an analytical target. To identify yellow chromophores, we employed ultra high-performance liquid chromatography (UHPLC) coupled with a photodiode array (PDA) detector and high-resolution mass spectrometry (HRMS). This platform, known as UHPLC-PDA-HRMS, is capable of identifying target analytes in complex mixtures. The PDA detector detects the presence of molecules of interest based on their absorption of ultraviolet (UV) or visible light. In this case, we isolated the peak that exhibited the maximum absorption of blue light, which means the most pronounced reflection of yellow light. Hence, this peak represents the most intense yellow chromophore within the context of the human epidermis. HRMS is essential to identify molecules of interest with high confidence, particularly in the absence of standards. This platform allows direct correlation between the detection and identification of the yellow chromophore in the native human epidermal explants. Leveraging this cutting-edge analysis, we successfully discovered the primary yellow chromophore, AGEY, in native human epidermal explants from a few donors (Figure 1). We further assessed the contribution of AGEY to skin yellowness using 14 skin tissue samples and confirmed a strong positive correlation between skin yellowness and AGEY intensity. The Pearson correlation coefficient (R) was found to be 0.72, with statistical significance (*p* = 0.0036). This finding strongly suggests that AGEY is the major contributor to the yellow color of the skin (Figure 3).

Interestingly, during our meticulous literature search, we coincidentally came across a paper stating that AGEY is one of the most intense yellow glycation chemicals observed in the food industry [25]. In the paper, the author utilized an artificial food glycation model and identified three other intense yellow-red colored chemicals, which are (a) 2-[2-furyl)methylidene]-4-hydroxy-5-methyl-2H-furan-3-one; (b) 2-[(2-furyl)methylidene]-4-hydroxy-5-[(E)-(2-furyl)methylidene]-methyl-2H-furan-3-one; and (c) (S)-4-[(E)-1-formyl-2-(2-furyl)ethenyl]-5-(2-furyl)-2-[(E)-(2-furyl)methylidene]-2,3-dihydo-α-amino-3-oxo-1H-pyrrole-1-acetic acid. However, in our experiments, we did not detect any of these three chromophores from native human explants, implying that AGEY may be the primary yellow glycation chemical in human skin. Through the utilization of a human epidermal explant model and induction of glycation using GLA, we confirmed that glycation indeed plays a role in driving the formation of AGEY and the elevation of skin yellowness (Figure 4), with evidence of a very high Pearson correlation coefficient (R = 0.77). Although the precise mechanisms underlying the formation of AGEY in human skin require further elucidation, our findings have important implications for the development of targeted interventions aimed at reducing chronic skin yellowness by inhibiting glycation pathways.

To identify effective solutions, we utilized a simple chemical artificial glycation platform employing gelatin and GLA. This model allowed us to identify four unique cosmetic ingredients: niacinamide, white water lily extract, lactobionic acid, and artichoke leaf extract. These ingredients are known to possess additional beneficial functions for the skin. For instance, niacinamide is recognized as a well-known skin lightening compound [28]. White water lily extract was reported to remove CML, which is one of the most abundant AGEs in the skin, by stimulating autophagy in keratinocytes [7]. Lactobionic acid is a polyhydroxy acid that is reported to provide skin exfoliation benefit without impairing the skin barrier or causing skin irritation [29]. Artichoke leaf extract is well known for its antioxidant properties and is reported to improve skin texture. We discovered that these cosmetic ingredients also possess anti-glycation effects and, when combined, they exhibit an even stronger effect in inhibiting glycation. We further confirmed that the combinations of the four cosmetic ingredients significantly suppressed AGEY formation and reduced skin yellowness in RHE models (Figure 6). Although further clinical validation in humans is required, our in vitro results implied that the combination might be an effective intervention to alleviate unpleasant skin yellowness.

In conclusion, our multidisciplinary approach, combining modern analytical chemistry tools, biological models, and biochemical analyses, has led, for the first time, to the discovery of AGEY as the (possibly most) intense yellow glycated compound in human skin. We have demonstrated in the skin explant model that AGEY is indeed formed through glycation and that its abundance is strongly correlated with the yellowness of skin tissues, confirming that AGEY is a critical factor in skin yellowness appearance and could be a barometer of skin glycation. Additionally, we have shown that a combination of four identified cosmetic ingredients can effectively inhibit the formation of AGEY while reducing yellowness in in vitro RHE models, which warrants further human clinical validation. As further investigations continue to unravel the chemical mechanisms involved in the formation of this novel yellow chromophore, we move closer to the targeted precision of treatments.

## 4. Materials and Methods

### 4.1. Chemicals, Reagents, and Cell Lines

Cell culture media and supplements were purchased from Thermo Fisher Scientific (Waltham, MA, USA), including EpiLife with 60 μM calcium (Cat. No. MEPI500CA), gentamicin/amphotericin B (500X; Cat. No. 50-0640), HKGS (100X; Cat. No. S-001-5), trypsin/EDTA solution (TE; Cat. No. R001100), trypsin neutralizer solution (TN; Cat. No. R002100), penicillin/streptomycin (10,000 U/mL, 100X; Cat. No. 15140122), and DPBS (Cat. No. 14190250). ViaStain AO/PI staining solution was purchased from Nexcelom Bioscience (Cat. No.: CS2-0106-5 mL, Lawrence, MA, USA). AccuGene 1× PBS was purchased from Lonza (Cat. No. 51225, Alpharetta, GA, USA). Other chemicals used were purchased from Sigma (St. Louis, MO, USA), including DMSO (Cat. No. D8418-100 mL), gelatin solution (Cat. No. G1393-100ML), DL-glyceraldehyde (GLA) (Cat. No. G5001), D-(+)Xylose (Cat. No. X3877-25G), L-alanine (Cat. No. 05129-25G), 2-furaldehyde (Cat. No. 185914-100ML), sodium sulfate anhydrous (Cat. No. 239313), ethyl acetate (Cat. No. 1.03649.000), and protease from streptomyces griseus (Pronase, Cat. No. P5147).

### 4.2. Characterization and Relative Quantification of Intense Yellow Chromophores in Human Epidermal Explant Tissue

Skin explant tissues were obtained from multiple surgical centers through an IRB-approved protocol reviewed by Advarra (Columbia, MD, USA), with annotation of donor age and Fitzpatrick skin type. The skin, post fat removal, was divided into 1.25 cm^2^ squares, immersed in 1 M NaCl with 10× penicillin/streptomycin (Invitrogen) and incubated at 37 °C overnight. The next day, the epidermis was detached using forceps, preserved in phosphate-buffered saline (PBS) supplemented with 1x penicillin/streptomycin, and stored at 4 °C until use, as outlined by Bachelor et al. [30]. The surface images of tissues were captured using a SpectroShade Micro spectrophotometer (SpectroShade USA, Oxnard, CA, USA). Yellowness scores (b*-value) were rendered from the captured images using the device’s built-in software.

Skin Epidermis was hydrolyzed with 6 N HCl at 110 °C for 16 h. The hydrolysate was then dried down and reconstituted with purified water prior to analysis using UHPLC-PDA-HRMS. Chromatographic separation was performed on an ACQUITY UPLC BEH Amide Column (130 Å, 1.7 µm, 2.1 mm × 150 mm, Waters). The LC gradient and detection was carried out on a Vanquish UHPLC-PDA system coupled with a Q Exactive HF HRMS mass spectrometer (Thermo Fisher Scientific). The mobile phase in the gradient mode consisted of water containing 0.1% formic acid as Mobile Phase A, and acetonitrile containing 0.1% formic acid as Mobile Phase B (90%B to 45%B in 18 min). The total running time was 30 min and the injection volume was 5 µL. The wavelengths of the PDA detector ranged from 190 to 680 nm. HRMS was operated under positive electrospray ionization (ESI+) and in full scan mode (*m*/*z*: 120 to 1500). The exact mass of the peaks present in a UV-Vis trace of 440 nm was obtained for peak identification. Relative quantification of the target peaks was performed by calculating the peak intensity in the extract ion chromatography (EIC).

### 4.3. Impact of GLA Treatment on AGEY Intensity and Skin Yellowness in Human Epidermal Explant Tissue

To assess whether AGEY can be formed by glycation, individual 6 mm punches of epidermal explant tissue (donor: 40 yrs, skin type IV) were incubated on inserts placed over the wells of 6-well culture plates. These tissues were treated with glycation reagent, GLA, at 5 mM, 10 mM, and 25 mM concentrations, respectively, diluted in phosphate-buffered saline (DPBS, Thermo Fisher Scientific). Treatment was triplicated for each condition. After 133 h GLA treatment, tissues were harvested for AGEY intensity quantification in the same way as described above. During GLA treatment, surface images of cultured tissue were captured using a SpectroShade Micro spectrophotometer (SpectroShade USA, Oxnard, CA, USA) at 20, 43, and 133 h time points. Yellowness scores (b*-value) were rendered from the captured images using the device’s built-in software.

### 4.4. Human Keratinocyte Cell Cultures and Preparation for AGEY Detection

Primary adult human keratinocytes from female donors (lots 1488, 1502, 2415, 2711, 2761) aged between 40 and 50 years old were purchased from Cell Applications (San Diego, CA, USA) and initially matured in EpiVita Serum-Free Growth Medium (Cell Applications) with Adult Keratinocyte Growth Supplement plus insulin and hydrocortisone (Cell Applications) and antibiotic-antimycotic solution (100×, Thermo Fisher Scientific). Once adult cells were established in T-75 flasks, a stepwise medium transition was conducted to EpiLife with calcium-supplemented HKGS (Thermo Fisher Scientific) and antibiotic-antimycotic solution. The medium was transitioned in 25% increments daily until cells were cultured in a 100% EpiLife medium. All cells were incubated at 37 °C under 5% CO_2_ and 95% humidity in T-75 flasks until 80–90% confluent. Cells were harvested via trypsinization, centrifuged to remove supernatant and obtain cell numbers, and stored at −80 °C until analysis. For relative AGEY quantification, 10 mg/mL pronase solution was prepared in 1x PBS buffer. A total of 0.3 mL of the pronase solution was added to each cell pellet. The cell pellet samples were incubated at 37 °C for 24 h with shaking and then centrifuged at 4500 rpm for 30 min at −4 °C. The supernatants obtained were analyzed for AGEY intensity quantification by HILIC-MS/MS, as described below.

### 4.5. Relative AGEY Quantification in Keratinocyte Cells Using HILIC-MS/MS Method

A reference material for AGEY was prepared following a scaled down procedure described previously [25]. Briefly, a solution of D-xylose (0.825 mmol) and L-alanine (0.2 mmol) in 10 mL of phosphate buffer (1 mM, pH 7.0) was heated under reflux for 10 min. Then, 2-Furaldehyde (0.125 mmol) was added, and heating was continued for another 60 min. After cooling to room temperature, the aqueous solution was extracted with ethyl acetate (5 × 5 mL). The organic layers were combined and dried over anhydrous sodium sulfate until they remained solid. Multiple attempts were made to purify AGEY for absolute quantification; however, these were without success. Therefore, we leveraged the reaction mixture to tune the target AGEY for method development with hydrophilic interaction chromatography (EMD ZIC-HILIC column, 2.1 mm × 150 mm) coupled with tandem mass spectrometry (Sciex 6500). The LC gradient and detection was carried out on a Shimadzu UFLC system. The mobile phase in gradient mode consisted of water containing 0.1% formic acid as Mobile Phase A, and acetonitrile containing 0.1% formic acid as Mobile Phase B (70%B to 10%B in 2.5 min). The total running time was 6 min and the injection volume was 5 µL. Three precursor-to-product ion transitions were monitored including (*m*/*z*: 313.4 to 295.3; 313.4 to 267.2; 313.4 to 80.9). Relative quantification of AGEY was performed based on the peak area counts in HILIC-MS/MS.

### 4.6. Gelatin-Based Chemical Glycation Assay as a Fast Compound Screening Tool

A simple and fast screening tool was developed by leveraging a GLA as a reducing agent and a mammalian gelatin (a hydrolyzed collagen protein) as a protein substrate. The AGEs that were formed could be detected by measuring the fluorescence intensity (“FLR”) or the yellow color formation assessed by b*-value. Lower FLR values or b*-values correspond to the formation of fewer AGEs, and thus less glycation.

Briefly, three replicates of each group were prepared in a 96-well plate (e.g., FALCON brand, REF 353072) with a reaction volume of 250 µL/well. With this setup, negative control wells consisted of gelatin at a concentration of 9 mg/mL diluted in DPBS buffer. Positive control wells contained gelatin at 9 mg/mL in DPBS buffer plus 40 mM of GLA. To the testing wells, test materials were introduced at the desired concentrations. Tested materials were sourced accordingly: aminoguanidine (Sigma), niacinamide (Sigma), white water lily extract (Glycoxyl^®^; Silab, Saint-Viance, France), lactobionic acid (Thermo Fisher Scientific), and artichoke leaf extract (Ichimaru Pharcos, Gifu, Japan). To maintain optimal conditions, plates with test samples were incubated at 37 °C, 5% CO_2_, and 95% humidity. FLR and b*-values were measured by the spectrophotometer (SPECTRAMAX Plus, Molecular Devices, San Jose, CA, USA) as an indicator of glycation crosslink formation at 0 h and approximately 20–24 h after the incubation started. For FLR, the spectrophotometer settings were 400/465 nm (ex/em). For yellowness (b*-value), the spectrophotometer collected the absorbance spectra from 350 nm to 750 nm at 10 nm intervals, and the collected spectra were converted to L*a*b* values. Changes in fluorescence intensity (∆FLR) or yellowness (∆b*) were calculated by subtracting baseline (time 0 h) values.

### 4.7. Effect of Anti-Glycation Compounds on AGEY Intensity and Skin Yellowness in 3D RHE Model

Commercially available 3D RHE models (EpiDerm^TM^) were procured from the MatTek Corporation (MatTek, Ashland, MA, USA) and cultured according to the manufacturer’s guidelines. Upon receipt, 3D RHE models were promptly placed in phenol red-free medium (Part No. EPI-100-ASY-PRF) and incubated at 37 °C with 95% humidity and 5% CO_2_ for 20 h to establish equilibrium. Afterwards, the cultures were treated with either 0.5 mM GLA only or 0.5 mM GLA with test compounds in the culture medium. All cultures were incubated with treatment at 37 °C with 95% humidity and 5% CO_2_ for 24 h. At the end of the incubation period, each culture was dissected from the tissue insert, rinsed with sterile water to eliminate any residual treatment, followed by peeling off the polycarbonate base membrane. Subsequently, the cultures were imaged using a handheld imaging device; the SpectroShade Micro spectrophotometer (SpectroShade USA, Oxnard, CA, USA). The built-in software was utilized to render b*-values from these images. After imaging, each culture was stored in a clear 1-dram glass vial on dry ice and later stored in a −80 °C freezer until subsequent analytical quantification of relative AGEY intensity using HILIC-MS/MS, as described above.

### 4.8. Statistical Analysis

Statistical significance for all experiments was determined by Student’s *t*-tests, unless stated otherwise. Pearson’s correlation analysis was used to determine the significance of the correlations between AGEY intensity and the b*-values for GLA treatment in human epidermal explant tissue. Values of *p* ≤ 0.05 were considered statistically significant.

## Figures and Tables

**Table 1:** Anti-glycation efficacy of selected cosmetic compounds via chemical glycation model.

Material **	Glycation % vs. GLA-Treated Control ***
Negative control (no GLA treatment)	0%
Positive control (GLA-treated, no compound treatment)	100%
Aminoguanidine hydrochloride (positive benchmark)	30% *
Quad combination of (a) to (d)	23% *
(a) Niacinamide	65% *
(b) White water lily extract	85% *
(c) Lactobionic acid	75% *
(d) Artichoke leaf extract	92% *

* *p* < 0.05 vs. positive control group. n = 3 per group. Student’s *t*-tests were performed on the absolute data of each ingredient and positive control set. ** Aminoguanidine was tested at 10 mM. Compounds were tested at (a) 5%, (b) 0.4%, (c) 0.1%, and (d) 0.1%. *** Glycation % = (ingredient—negative control)/(positive control—negative control) x 100 using fluorescent measurement data.

## Data Availability

The data presented in this study are available on request from the corresponding author.
